# The Relationship between Sleep Complaints, Depression, and Executive Functions on Older Adults

**DOI:** 10.3389/fpsyg.2016.01547

**Published:** 2016-10-07

**Authors:** Katie M. de Almondes, Mônica V. Costa, Leandro F. Malloy-Diniz, Breno S. Diniz

**Affiliations:** ^1^Group of Research Neuroscience Applied, Basic Process and Chronobiolog, Department of Psychology, Federal University of Rio Grande do NorteNatal, Brazil; ^2^Laboratory for Investigations in Clinical Neuroscience, School of Medicine, Federal University of Minas GeraisBelo Horizonte, Brazil; ^3^Department of Mental Health, School of Medicine, National Institute of Science & Technology Molecular Medicine, Federal University of Minas GeraisBelo Horizonte, Brazil; ^4^Department of Psychiatry and Behavioral Sciences, University of Texas Health Science Center at HoustonHouston, TX, USA

**Keywords:** sleep, executive function, depression, geriatrics, mild cognitive impairment, older adults, neuropsychological tests

## Abstract

**Aim:** In this manuscript, we report data on the association between executive functions screened by Frontal Assessment Battery, Five Digit Test and Digit Span with self-reported depressive symptoms and sleep complaints in non-demented older adults.

**Methods:** A total sample of 95 non-demented older adults performed Geriatric Depression Scale short version, Frontal Assessment Battery, Five Digit Test, Digit Span, and clinical interview. We split participants in groups stratified by age according to: young-old (60–69 years of age), old-old (70–79 years), and oldest-old (>80 years) and compared these three groups on the sociodemographic characteristics and executive functions performance. We carried out Poisson regression with robust error variance to verify sleep complaints and depression effects on executive functions performance. Gender, age, years of formal education, use of antidepressants and of benzodiazepines were considered as confounding variables, taking into account executive functions as dependent and sleep complaints and depression as independent variables.

**Results:** Controlling the effect of age, gender, years of formal education, use of benzodiazepines and of antidepressants there was a significant influence of depression in motor programming, inhibitory control, and working memory. Individuals without depression show motor programming scores 68.4% higher, inhibitory control scores 3 times greater and working memory scores also 3 times greater than individuals without depression. There was a significant influence of sleep complaints in phonemic fluency, motor programming, inhibitory control, and working memory. Individuals without sleep complaints show phonemic fluency scores 2 times greater than, motor programming scores 85.9% higher, inhibitory control scores 3 times greater and working memory scores also 3 times greater than individuals without sleep complaints.

**Conclusions:** Sleep complaints are associated with phonemic fluency, motor programming, inhibitory control, and working memory impairment. Depression symptoms presence are associated with motor programming and working memory performances. Depression and sleep complaints interaction would determine worse phonemic fluency, inhibitory control and working memory cognitive performance than these two conditions alone.

## Introduction

Across the lifespan, sleep complaints are often comorbid with psychiatric disorders and are common in depression. Sleep has been studied extensively in this psychiatric disorder and were found objective, robust and relatively specific changes in sleep architecture related to neurobiology of depression (Benca and Peterson, 2008). On the present study, we provide a solid background for the importance of taking into account the interaction between cognition, sleep disorders, and depression among older adults.

During a depressive episode, cortical metabolic activity is increased in frontal areas, what impact sleep and cause subjective complaints of non-restorative sleep. In depressed patients, there is an imbalance of interaction between cholinergic and adrenergic mechanisms that promote abnormalities in sleep architecture (non-REM and REM). There are changes in non-REM sleep that result in excessive daytime sleepiness. Moreover, depressed patients exhibit cortisol and temperature desynchronization on advanced sleep phase (the expression of these rhythms appears earlier than expected) and on the sleep-wake cycle (Cajochen et al., 2001).

Processing speed (Bastien et al., 2003), executive functions such as switching, working memory, problem solving and inhibitory control (Nebes et al., 2009) and memory (Haimov et al., 2008), are associated with self-reported sleep complaints. Moreover, sleep disorders as self-reported change in sleep increases the risk of cognitive decline (Hahn et al., 2014).

Depression also increases susceptibility to cognitive decline and neurodegenerative dementia (Diniz et al., 2013), impact activities of daily living and patient quality of life, and rising health care costs. Among cognitive impairment associated with depression, executive function is the most prominent (Snyder, 2013), but deficits in processing speed (Jungwirth et al., 2011), spatial processing (Elderkin-Thompson et al., 2004), and verbal episodic memory (Nebes et al., 2000) are also found. They mediate decline in more complex functions such as attention, planning and problem solving. There is evidence of a close relationship between sleep loss, depression and cognition with effect sizes in “moderate range” (Lim and Dinges, 2010).

The aim of this study was to investigate the association between executive functions screened by Frontal Assessment Battery, Five Digit Test and Digit Span with comorbid self-reported depressive symptoms and sleep complaints in non-demented older adults. Overall, we hypothesized that sleep complaints and depression should be associated with executive functions impairment and that depression and sleep complaints interaction would determine a worse cognitive performance than these two conditions alone.

## Materials and methods

### Participants

We included a convenience sample of 95 older adults that were consecutively assessed at geriatric outpatient clinic at Belo Horizonte, Brazil between February 2011 and December 2015. These participants were visiting the clinic as a center of specialized geriatric treatments referred by other smaller primary care centers. The inclusion criteria were: age of 60 years or older. We excluded individuals that satisfied DSM-V and consensus criteria for dementia as NINCDS-ADRDA; individuals with major psychiatric comorbidities (e.g., bipolar disorder, schizophrenia, substance abuse); sensory disabilities that interfere with cognitive evaluation; neurodegenerative diseases of the central nervous system diagnosis (e.g., Parkinson's disease); clinical or historical evidence of transient ischemic attack or stroke or clinical instability. In the end, we have had a total of 175 participants. Of these 57 were excluded due to meet criteria for dementia due to Alzheimer's disease and 23 for dementia due to other etiologies such as frontotemporal and vascular dementia. Participants were instructed to perform tasks with the dominant hand. This study was approved by the local Ethics Committee (Belo Horizonte, MG) and all subjects agreed and signed the informed consent in accordance with the Declaration of Helsinki.

### Neuropsychological assessment

Neuropsychological assessments occurred between 08:00 a.m. and 05:00 p.m., then the time of day was not in a similar range for all the participants. There are evidence that cognitive performance may varies during the course of the day and reflect the level of alertness, which is determined by circadian rhythms (Schmidt et al., 2007). It is discussed that these findings can be attributed to underlying circadian and homeostatic factors. Different aspects of attention follow different time-of-day variations (Kraemer et al., 2000) and this bias was not controlled on the present study.

Diagnosis involved a clinical interview and a neuropsychological evaluation with a protocol previously validated for this population (de Paula et al., 2013a) composed by the following instruments: Mini Mental State Examination (MMSE) Brazilian version as a measure of general cognition; Clock Drawing Test as a measure of spatial processing, praxia, and executive functions; The Digit Span Task adapted from Wechsler Adult Intelligence Scale to assess short term memory; Corsi Blocks a measure of spatial short term memory; Semantic Verbal Fluency that measures the fluency component of the executive functions. Finally, Token Test-Short Version was used to assess language comprehension. Functional status was also assessed in the clinical interview using the caregivers' reports. Based on this evaluation, non-demented participants were invited for participation.

The MMSE were performed as a measure of general cognitive functioning and Activities of Daily Living (ADLs) inventory as a measure of functional status. Frontal Assessment Battery (FAB) Brazilian version was used to access executive functions. It presents a good validity for this population (de Paula et al., 2013b) and consists of six subtests which measures: Categorization, verbal fluency, motor programming, sensitivity to interference, inhibitory control, and environmental autonomy.

### Depressive symptoms assessment

All the participants performed the short version (15-items) of the Geriatric Depression Scale (GDS-15) and we considered the cutoff 6 (non-case/case) to classify presence/absence of significant depressive symptoms. To minimize low education bias, the scale was applied orally and the questions were repeated if requested by the participant. Depressive symptoms assessment was conducted on the same date of neuropsychological assessment and participant were required to answer according to how he felt in “last few weeks.”

### Sleep and pharmacological assessment

Sleep complaints was evaluated qualitatively through of a clinical interview with self report of participants. It was required to participants if they had noticed sleep-related problems in recent months or weeks to date. The response was evaluated in “yes/no” and they also describe the sleep problems.

The use of drugs, including benzodiazepines, was assessed by patients' medical records. Pharmacological treatments were evaluated during the anamnesis. Patients and caregivers reported which drugs were used at that time, and based on these data the drugs were classified as antidepressants or benzodiazepines. In the database the names of the drugs were specified, the number of medications in use and dichotomous variables were created for use of hypnotics and antidepressants.

### Statistical procedures

Once our data distribution was predominantly non-parametric, the statistical procedures were performed by non-parametric tests. Sociodemographic description and neuropsychological tests were performed through Mann–Whitney *U*-test for continuous variables and by Chi-Square for dummies variables.

We split participants in groups stratified by age according to: young-old (60–69 years of age), old-old (70–79 years), and oldest-old (>80 years) and we compared these three groups on the sociodemographic characteristics and executive functions performance through Kruskal–Wallis Test.

We carried out Poisson regression with robust error variance (McCullagh and Nelder, 1983) to verify depression and sleep complaints effects on executive functions performance. Gender, age, years of formal education use of antidepressants and of benzodiazepines were considered as confounding variables, taking into account executive functions as dependent and depression and sleep complaints, as independent variables. These statistical procedures were performed using SPSS 21.0. and statistical significance was established at 0.05.

## Results

The demographic description and neuropsychological tests performance are shown in Table 1 for Chi-square analysis and in Table 2 for Mann–Whitney. A total of 95 non-demented older adults $(60–91 years (*M* = 74, *SD* = 7), predominantly low formal education (*M* = 5, *SD* = 4) were enrolled in this study. In this sample, 36% were depressed, 48% with sleep complaints, 14% in use of benzodiazepines, and 23% of antidepressants including 15 participants in use of selective serotonin reuptake inhibitors (SSRI) and 7 in use of tricyclic antidepressant (TCA) (Table 1). No significant differences between age (*U* = 1128.00, *p* = 0.407), education (*U* = 1069.00, *p* = 0.273) or proportion of men and women (χ^2^ = 0.024, *p* = 0.524) were found between patients with and without sleep complaints. There is no difference between performance on Mini Mental State Examination between two conditions (*U* = 594.00, *p* = 0.756), daily function activities (χ^2^ = 0.437, *p* = 0.358), FAB total score (*U* = 1177.00, *p* = 0.623), or use of benzodiazepines (χ^2^ = 2.248, *p* = 0.113). Differences in presence of significant depressive symptoms classified through GDS-15 cutoff between groups with and without sleep complaints are found (χ^2^ = 16.429, *p* < 0.001) with more patients with depression in the group with sleep complaints.

**Table 1:** **Results of Chi-square Tests on gender, use of benzodiazepines and use of antidepressants proportion for participants with and without sleep complaints**.

	**Total sample (%)**	**Sleep complaints (%)**	**With no sleep complaints (%)**	**Groups comparison between participants with sleep complaints and without sleep complaints**	
				***X*^2^**	***p***
Gender^a^	62	73	77	0.332	0.564
Benzodiazepines^b^	12	20	10	2.248	0.134
Antidepressants^c^	22	32	15	3.644	0.056
Depression	31	57	17	16.266	<0.001

a *Proportion of women*.

b *Proportion of use*.

c *Proportion of use*.

**Table 2:** **Group comparisons between participants with sleep complaints and without sleep complaints**.

	**Total sample**			**Sleep complaints**			**Without sleep complaints**			**Groups comparisons**		
	***M***	***SD***	**Range**	***M***	***SD***	**Range**	***M***	***SD***	**Range**	**MW-U**	***Z***	***p***
Age	74.46	7.355	58–91	73.80	7.654	58–90	75.08	6.990	62–91	1128	−0.829	0.407
Education	5.68	4.865	0–22	5.21	4.964	0–22	6.12	4.853	0–21	1069	−1.096	0.273
MMSE	25.67	3.078	15–30	25.08	4.041	15–30	25.87	2.419	21–30	594	−0.311	0.756
FAB	12.23	3.281	4–18	12.14	3.380	4–18	12.48	3.245	6–18	1177	−0.492	0.623
ADL	3.63	4.834	0–23	3.86	4.740	0–18	2.91	4.568	0–23	542	−1.201	0.23

Values are frequencies or Mean ± Standard Deviation (SD); FAB, Frontal Assessment Battery; MMSE, Mini-Mental State Examination; ADL, Activities of Daily Living; MW-U, Mann–Whitney U-Test; Z, difference between standard scores; p, significance level.

In Tables 3, 4 we could see descriptive statistics between age groups for socio-demographic variables and results of group comparisons between executive functions performance among non-demented elderly stratified by age. There are significant differences on categorization between young-old (60–69 years of age) and oldest-old (>80 years) (χ^2^ = 18.479, *p* = 0.046). Phonemic verbal fluency performance is significantly different between young-old (60–69 years of age) and old-old (70–79 years) (χ^2^ = 17.280, *p* = 0.019). In relation to motor programming there are differences between young-old (60–69 years of age) and old-old (70–79 years) (χ^2^ = 19.000, *p* = 0.031) and between young-old (60–69 years of age) and oldest-old (>80 years) (χ^2^ = 24.750, *p* = 0.003). Otherwise, performance on inhibitory control is only significantly different between old-old (70–79 years) and oldest-old (>80 years) (χ^2^ = 19.910, *p* = 0.006). For FAB total score, performance is significantly different between young-old (60–69 years of age) and old-old (70–79 years) (χ^2^ = 17.557, *p* = 0.032) and young-old (60–69 years of age) and oldest-old (>80 years) (χ^2^ = 32.250, *p* > 0.001).

**Table 3:** **Descriptive statistics between age groups for socio-demographic variables**.

	**Total sample**	**Age groups**		
**Age ranges**	**60–91**	**Young–old 60–69**	**Old–old 70–79**	**Oldest–old >80**
*N*	95	24	47	24
Age	74.49 ± 7.29	65.54 ± 2.637	74.62 ± 2.75	83.88 ± 3.180
**SEX**				
Men	29	5	16	8
Woman	66	19	31	16
**EDUCATION**				
Years of formal education	5.71 ± 4.89	7.142 ± 5.174	5.78 ± 5.133	4.08 ± 3.538
Illiteracy	7	2	3	2
Primary school	65	14	34	20
Secondary or Higher	23	8	10	2
Pfeffer scale	3.400 ± 4.648	4.230 ± 4.206	2.760 ± 3.940	4.050 ± 6.013
MMSE	25.591 ± 3.083	27 ± 2.221	25.588 ± 2.664	25.000 ± 3.293

Values are frequencies or Mean ± Standard Deviation (SD); MMSE, Mini Mental State Examination.

**Table 4:** **Results of group comparisons between executive functions performance among non-demented elderly stratified by age**.

	**Total sample**	**Age groups**			**Group comparisons**		
		**1—Young-Old**	**2—Old-Old**	**3—Oldest-Old**	***X*^2^**	***p***	**Differences between age groups**
**Age ranges**	**60–91**	**60–69**	**70–79**	**>80**			
FAB (total score)	12.32 ± 3.294	14.33 ± 2.353	12.34 ± 3.171	10.54 ± 3.148	17.557	0.032	2 > 3
					32.250	< 0.001	1 > 3
FAB—categorization	1.505 ± 1.071	1.958 ± 0.908	1.456 ± 1.110	1.208 ± 1.021	18.479	0.046	1 > 3
FAB—fonemic fluency	2.147 ± 0.989	2.625 ± 0.711	1.978 ± 1.022	2.083 ± 0.974	17.280	0.019	1 > 2
FAB—motor programming	1.726 ± 0.950	2.042 ± 0.806	1.782 ± 0.987	1.333 ± 0.917	19.000	0.031	1 > 2
					24.750	0.003	1 > 3
FAB—sensitivity to interference	2.357 ± 1.051	2.708 ± 0.751	2.326 ± 1.097	2.083 ± 1.176	2.988	0.131	–
FAB—inhibitory control	1.589 ± 1.180	2.000 ± 1.142	1.782 ± 1.153	0.875 ± 0.947	19.910	0.006	2 > 3
Five digit test inhibitory control	3.730 ± 5.535	2.940 ± 5.048	3.360 ± 5.709	5.110 ± 5.516	7.232	0.027	1 > 3
Five digit test cognitive flexibility	8.980 ± 8.611	7.330 ± 7.191	7.670 ± 7.680	12.860 ± 10.101	8.184	0.017	1 > 3
							2 > 3
Digit span backward	11.480 ± 9.573	13.920 ± 13.061	10.960 ± 8.565	10.570 ± 7.675	0.735	0.692	–

Values are frequencies or Mean ± Standard Deviation (SD); FAB, Frontal Assessment Battery.

In Table 5 we present the Poisson regression to verify depression and sleep complaints effects on executive functions performance. Controlling the effect of age, gender, years of formal education, use of benzodiazepines and of antidepressants sleep complaints are associated with cognitive impairment on phonemic fluency, motor programming, inhibitory control and working memory. Individuals without sleep complaints have FAB motor programming scores 85.9% higher, phonemic fluency scores twice as high as, inhibitory control scores three times as high as and Digit Span Backward scores three times as high as individuals with sleep complaints.

**Table 5:** **Effects of depression and sleep complaints on executive functions performance**.

		**Independent variables**							
**Dependent variables- neuropsychological assessment**	**Parameters**	**Age (years)**	**Formal education (years)**	**Gender = Female**	**Depression**	**Sleep complaints**	**Use of benzodiaze-pines**	**Use of anti-depressants**	**Depression/Sleep complaints interaction**
FAB conceptualization	exp(β)	0.981	1.053	1.228	1.311	1.699	1.384	1.052	0.672
	*p*-value	0.036^*^	0.000^*^	0.147	0.607	0.334	0.398	0.802	0.486
FAB phonemic fluency	exp(β)	0.997	1.032	1.137	1.812	2.095	0.808	0.966	0.434
	*p*-value	0.657	0.000^*^	0.151	0.116	0.049^*^	0.016^*^	0.785	0.034^*^
FAB motor programming	exp(β)	0.987	1.021	1.274	1.684	1.859	1.141	1.392	0.605
	*p*-value	0.130	0.040^*^	0.061	0.014^*^	0.028	0.450	0.023^*^	0.112
FAB sensitivity to interference	exp(β)	0.978	1.000	0.934	1.662	1.610	6.509	0.975	0.578
	*p*-value	0.009^*^	0.984	0.517	0.137	0.175	0.040^*^	0.804	0.134
FAB inhibitory control	exp(β)	0.964	1.017	1.377	2.796	3.240	1.093	1.754	0.250
	*P*-value	0.006^*^	0.318	0.090	0.053^*^	0.036^*^	0.888	0.062	0.021^*^
FAB environmental autonomy	exp(β)	1.000	1.002	0.972	0.960	1.002	1.151	1.004	1.039
	*p*-value	0.581	0.252	0.115	0.239	0.885	0.133	0.856	0.246
FAB total score	exp(β)	0.985	1.016	1.125	1.036	1.168	1.087	1.092	0.843
	*P*-value	0.000^*^	0.002^*^	0.017^*^	0.709	0.094	0.290	0.125	0.136
Five digit test inhibitory control	exp(β)	1.033	0.963	0.727	0.887	1.026	0.666	0.970	0.950
	*p*-value	0.169	0.170	0.320	0.824	0.969	0.479	0.924	0.943
Five digit test cognitive flexibility	exp(β)	1.035	0.969	1.009	0.725	0.900	1.037	0.718	0.878
	*p*-value	0.009^*^	0.118	0.963	0.285	0.733	0.883	0.105	0.736
Digit span backward	exp(β)	0.994	1.061	1.025	3.467	3.438	1.071	0.991	0.299
	*p*-value	0.607	0.000^*^	0.873	0.000^*^	0.000^*^	0.771	0.959	0.001^*^

*exp(β), exponentiated values of the coefficients; FAB, Frontal Assessment Battery*.

* *p-value < 0.05*.

Depression complaints is associated with motor programming and working memory impairment. Individuals without depression symptoms have FAB motor programming 68.4% higher and Digit Span Backward scores three times as high as individuals with depression symptoms.

Individuals without use of benzodiazepines presents sensitivity to interference scores six times as high as individuals in use of benzodiazepines. However, the use is associated with an increase of 19.2% on phonemic fluency scores. Individuals in use of antidepressants have motor programming scores 39.2% higher than individuals with no use of antidepressants.

Interaction between sleep and depression complaints is associated with phonemic fluency, inhibitory control and working memory impairment. Those which showed these two types of symptoms presents FAB fluency subtest scores 56.6%, FAB inhibitory control subtest scores 75% and Digit Span Backward scores 70.1% lower than individuals with no complaints.

Sociodemographic variables are also associated with executive functions performances. At each year added on age, FAB conceptualization scores decreases 1.9%, sensitivity to interference scores decreases 2.2%, inhibitory control scores decreases 3.6%, FAB total scores decreases 1.5% and errors on Five Digit Test cognitive flexibility task increases 3.5%. At each year added on formal education, FAB conceptualization scores increases 5.3%, phonemic verbal fluency scores increases 3.2%, motor programing scores increases 2.1%, FAB total scores increases 1.6% and Digit Span Backward scores increases 6.1%. Female individuals have FAB total scores 1.7% higher than the males (Table 5).

## Discussion

Sleep complaints are associated with phonemic fluency, motor programming, inhibitory control, and working memory impairment. Depression symptoms presence are also associated with motor programming and working memory performances. Depression and sleep complaints interaction would determine phonemic fluency, inhibitory control, and working memory worse cognitive performances than these two conditions alone.

We found that depression is associated with less efficiency on motor programming. According to previous studies left dorsal prefrontal cortex and the right anterior cingulate cortex are more involved when subjects paid attention to the performance of a sequence of movements in comparison to automatic performance of this same task (Jueptner et al., 1997). Automatic aspects of motor performance are associated with subcortical structures, but sequential actions tasks with prefrontal networks (Fuster, 2001). It suggests that preparation of motor responses and learning of sequences of movements differs from an automatic condition and demands more from areas associated with executive function, which could be the same regions involved in depression. Studies of motor performance in depression corroborates these findings showing that cognitive difficulties increased with complexity of the tasks (Sabbe et al., 1996) and could be affected by pharmacological treatment (Mergl et al., 2007).

Long-term use of hypnotics may cause poor sleep quality (Vignola et al., 2000). It is also known that the use of benzodiazepines by older adults leads to cognitive decline (Glass et al., 2005). On the present study the use of benzodiazepines is associated with sensitivity to interference impairment. This is a component of executive functions related to selective attention measured by a task of conflicting instructions. Previous studies have also found association between attention and use of benzodiazepines (Barker et al., 2004) that could be due to inductive sedation effect (Chennu and Bekinschtein, 2012). Individuals in use of benzodiazepines showed phonemic fluency scores higher than individuals that are not in use of benzodiazepines. Considering that individuals without sleep complaints present phonemic fluency scores twice as high as those with sleep complaints, this may reflect the differences between those treated and untreated sleep disorders individuals. Those individuals treated probably have an improvement on fluency performance in comparison to untreated. Individuals in use of antidepressants also show motor programming scores higher than individuals with no use of antidepressants. Probably, this may reflect the differences between those treated and untreated individuals.

Deficits in verbal fluency are common finding after sleep loss, with reports of fewer words, and perseveration of incorrect responses, on tasks of verbal fluency (Harrison and Horne, 1998). These findings were not replicated in the study of (Tucker et al., 2010), but results have suggested an association between subjective sleep complaints and decline in phonemic fluency performance. Recently, McGregor and Alper (2015) showed that adults with sleep disorders were at a higher risk for language problems than healthy sleepers. The language problems typically co-occurred with problems of attention and executive function.

The phonemic fluency is considered a complex task related to cognitive flexibility, sustained attention, inhibitory control, and semantic memory (Shao et al., 2014). All these processes for which sleep complaints determined lower scores are cognitive abilities controlled and can be considered executive functions, especially working memory and inhibitory control. Controlled processes as attention and working memory are linked to the functioning of frontal lobes (Alhola and Polo-Kantola, 2007). Whereas, the frontal areas are vulnerable to sleep disorders (Harrison et al., 2000), this can be associated with impairment in inhibitory control, working memory, and verbal fluency in individuals with sleep complaints. Another related issue is the developmental process of the executive functions. The functional apex of executive functions is reached during adulthood and from this point is expected to decline (Goh et al., 2013). The course of development these abilities are therefore associated with a curve in the inverted U shape, so such impairments are expected on older adults.

This sample was composed by low formal education older adults due to the characteristics of the origin country. Educational level is associated with cognitive decline (Ardila et al., 2000) and is considered a main factor throughout cognitive aging. Performance on FAB is influenced by formal education (Beato et al., 2012; de Paula et al., 2013a) age. In our sample formal education is associated with motor programming, FAB total scores increases, working memory, categorization, and phonemic verbal fluency. In young old percentile for illiterates is significantly different from university graduates (de Paula et al., 2013a). The scale validation study for this population (Beato et al., 2012) suggest four formal education tracks, with 50th percentile varying significantly according to the formal educational level.

The differences between age groups in performance on the FAB subtests are in agreement with previous studies which indicate a decline in executive functions over the years (Salthouse et al., 2003) even on healthy aging. Previous studies also found no age-related deficits specific to selective attention or task-switching location (Verhaeghen and Cerella, 2002). At each year added on age, conceptualization task scores decreases 1.9%, sensitivity to interference scores decreases 2.2%, inhibitory control scores decreases 3.6%, FAB total scores decreases 1.5%, and cognitive flexibility task errors increases 3.5%.

Finally, the study should be viewed in the light of these limitations: The pharmacological treatments as with antidepressants have not been completely controlled; convenience sample with inherent problems of selection. Outpatient clinic samples tend to present higher prevalence of cardiovascular diseases and other conditions increased by aging compared to the general population. Some confounders like these vascular risk factors or diseases were not controlled. The sleep evaluation of was not performed by a sleep specialist and the sleep quality evaluation depends on self report. It is known that depressed patients tend to overestimate or underestimate their sleep problems in self reports; The use of FAB to evaluate executive functions is also limited due to being a screening test and because items are evaluated on a scale between 0 and 3 points, which reduces the variation range. Additionally, Outpatient clinic samples tend to present higher prevalence of cardiovascular diseases and other conditions increased by aging compared to the general population. Some confounders like these vascular risk factors or diseases were not controlled.

Our findings suggest that depression and sleep are associated with impairment on executive functions in non-demented older adults, and that use of benzodiazepines and educational level are important variables. These data are important because executive functions are predictors of dementia in older adults.

## Author contributions

All authors contributed and participated in the preparation of the article and all research steps: KMA contributed to the study on conception and design, on the statistical analysis, interpretation of data, wrote this article and approved the final version of the manuscript; MC contributed with acquisition of data, on supervising the geriatric assessment of the participants, on the statistical analysis, manuscript writing, and approved the final version of the manuscript; LM helped to design the study, on supervising the geriatric assessment of the participants, reviewed the statistical analysis and the manuscript and approved the final version of the manuscript; BD contributed to the study on conception and design, reviewed the statistical analysis and the manuscript, and approved the final version of the manuscript.

## Funding

The present study was supported by Grants: CNPq 484609/2012-2 and CNPq 471614/2014-9 (KMA); CBB-APQ-00075-09, APQ-01972/12-10, APQ-02755-10, APQ-04706-10 from FAPEMIG, and 573646/2008-2 from CNPq (LM); CNPq 472138/2013-8 and CNPq 466623/2014-3 (BD).

### Conflict of interest statement

The authors declare that the research was conducted in the absence of any commercial or financial relationships that could be construed as a potential conflict of interest.
